# Chimeric virus-like particles containing influenza HA antigen and GPI-CCL28 induce long-lasting mucosal immunity against H3N2 viruses

**DOI:** 10.1038/srep40226

**Published:** 2017-01-09

**Authors:** Teena Mohan, Zachary Berman, Yuan Luo, Chao Wang, Shelly Wang, Richard W. Compans, Bao-Zhong Wang

**Affiliations:** 1 Center for Inflammation, Immunity & Infection, Institute for Biomedical Sciences, Georgia State University, 100 Piedmont Ave SE, Atlanta, GA 30303, USA; 2 Department of Microbiology & Immunology, School of Medicine Emory University, 1518 Clifton Road, Atlanta, GA 30322, USA

## Abstract

Influenza virus is a significant cause of morbidity and mortality, with worldwide seasonal epidemics. The duration and quality of humoral immunity and generation of immunological memory to vaccines is critical for protective immunity. In the current study, we examined the long-lasting protective efficacy of chimeric VLPs (cVLPs) containing influenza HA and GPI-anchored CCL28 as antigen and mucosal adjuvant, respectively, when immunized intranasally in mice. We report that the cVLPs induced significantly higher and sustainable levels of virus-specific antibody responses, especially IgA levels and hemagglutination inhibition (HAI) titers, more than 8-month post-vaccination compared to influenza VLPs without CCL28 or influenza VLPs physically mixed with sCCL28 (soluble) in mice. After challenging the vaccinated animals at month 8 with H3N2 viruses, the cVLP group also demonstrated strong recall responses. On day 4 post-challenge, we measured increased antibody levels, ASCs and HAI titers with reduced viral load and inflammatory responses in the cVLP group. The animals vaccinated with the cVLP showed 20% cross-protection against drifted (Philippines) and 60% protection against homologous (Aichi) H3N2 viruses. Thus, the results suggest that the GPI-anchored CCL28 induces significantly higher mucosal antibody responses, involved in providing long-term cross-protection against H3N2 influenza virus when compared to other vaccination groups.

Influenza is a serious respiratory disease spread around the world, causing seasonal epidemics and recurrent outbreaks^1^,^2^. The influenza virus is a significant cause of morbidity and mortality worldwide resulting in over 200,000 hospitalizations and approximately 36,000 annual deaths in the United States alone^3^. Successful vaccines are the mainstay of efforts to reduce the substantial health burden inflicted by the virus. Vaccination against the influenza virus is currently the most efficient and economical way of reducing the number of infections. Conventional influenza vaccines have various limitations such as reduced efficacy in some populations, antigenic diversity, slow production time, and manufacturing restrictions^4^,^5^. Current vaccine pipelines and strategies must improve immune responses to vaccines, especially in various at-risk target populations, improve the manufacturing processes, increase the cross-reactive immunogenicity, and develop a new-generation of vaccines with long-lasting immunity^6^,^7^. The creation of a cost-effective and universal influenza vaccine has been one of the leading public health issues of the last several decades. However, such a vaccine has remained elusive to this day. The success of current influenza vaccine campaigns depends heavily on a more scalable platform with low cost that can induce long-term cross-protective immunity. The bottlenecks of conventional vaccines encouraged us to design a next-generation influenza vaccine which can be produced in a non-infectious, egg-independent manner and elicit long-lasting broadly cross-reactive immunity.

Virus-like particles (VLPs) are rapidly manufactured, hollow-core, non-infectious virus particles which present structurally native, immunologically relevant viral antigens^8^,^9^. The ease of manipulation of the VLP composition is a major advantage of this platform. Influenza VLPs, as a promising vaccine candidate, have been shown to induce high neutralizing antibody titers, strong protective immunity, and also activate innate immunity *via* pathogen recognition receptors^10^,^11^,^12^,^13^.

It should be emphasized that several unique vaccine strategies are being developed to induce protective mucosal immunity. The mucosal immune system represents the first line of immunological defense against pathogens encountering the mucosal surfaces of the body. The influenza virus enters through the respiratory tract; therefore, the mucosal antiviral responses such as local innate and IgA responses are thought to contribute as a first line of defense in immunity. Since local IgA responses have been shown to play an important role in responses to natural infection and also to be involved in cross-protection, the research on mucosal influenza vaccines continues to expand. In various experimental settings, passive local transfer of antigen-specific IgA from immunized to naïve mice, protected the animals when challenged with homologous or drifted influenza viruses^14^,^15^. Several studies in mice showed induction of strong cross-protective immunity through IgA antibodies^16^,^17^,^18^. During immune exclusion, the pre-existing secretory IgA (S-IgA) antibodies can provide immediate immunity through the elimination of the pathogen before it even passes the mucosal barrier and enters the body^19^,^20^. Thus, it would be advantageous to develop a next-generation mucosal influenza vaccine.

Successful vaccines against influenza rely on the generation of long-lasting antibodies that are able to rapidly neutralize an invading virus and thus prevent infection in immunized individuals. Although seasonal influenza vaccines can effectively prevent infection and outbreaks of matched viruses during a particular season, these vaccines do not provide long-term protection and people can still become infected after vaccination^21^. The current challenge in influenza vaccine design is to induce long-lasting cross-protective immune responses against homologous, drifted, or shifted strains. Immune memory signatures including T cell and antibody responses are the key parameters for inducing such protection^22^. Memory cells are long-lived and respond rapidly against the same pathogen in subsequent infections. Antibody persistence, duration and quality of produced antibodies, and generation of immunological memory are required for long-term protective immunity^23^,^24^. Thus, influenza vaccines that can elicit efficient cross-protection with the induction of memory cells and neutralizing antibodies may protect humans effectively from subsequent influenza infections.

In order to increase the efficacy of any vaccine in regards to long-lasting immunity, adjuvants may be essential. CCL28 (mucosae-associated epithelial chemokine, MEC) is a CC chemokine, which binds to CCR3 and CCR10 chemokine receptors and has been shown in numerous studies to be involved in the migration of antibody secreting cells (ASCs) into mucosal tissues^25^,^26^,^27^. In particular, CCL28 attracts IgA but not IgG or IgM producing cells and also promotes their migration to different mucosal sites^28^,^29^,^30^. Because of its specific role in orchestrating the localization of IgA ASCs at mucosal sites, we previously analyzed the adjuvanticity of GPI-anchored CCL28 co-incorporated into influenza VLPs. We demonstrated that GPI-CCL28 in influenza VLPs acts as a strong immunostimulator at both systemic and mucosal sites, boosting significant cross-protection in animals against heterologous viruses across a large distance^31^.

In the current approach, we present influenza HA antigen on VLPs in an immunogenic conformation together with GPI-anchored CCL28, an adjuvant capable of enhancing antigen-specific long-lived protective immunogenicity against influenza H3N2 viruses.

## Results

### Influenza VLPs containing GPI-anchored CCL28 vaccine formulations elicited high and sustained levels of long-lived virus-specific antibodies

**Figure 1:**
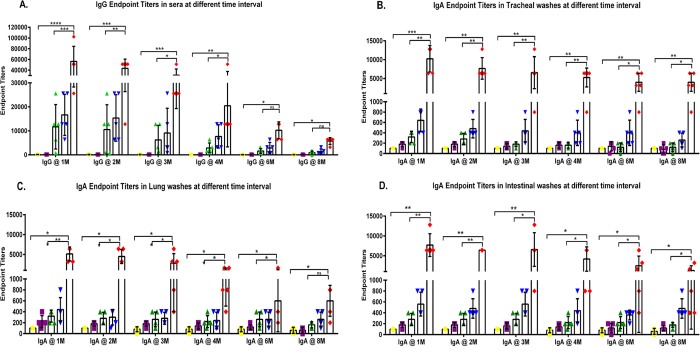
Virus-specific IgG and IgA endpoint titers. Figure represents influenza Aichi virus-specific antibody mean endpoint titers in (**A**) Sera, (**B**) Tracheal, (**C**) Lung, and (**D**) Intestinal washes. Mice were immunized with PBS, M1, HA/M1, HA/M1 with sCCL28, and HA/M1/GPI-CCL28 VLPs at day 0; and a booster dose was given on day 14 with the same formulations. Blood and mucosal washes were collected at months 1, 2, 3, 4, 6, 8 and antibody titers were investigated by ELISA using the inactivated Aichi virus as the coated antigen. The highest dilution which gave an OD_450_ two fold higher than that of the naive group without dilution, was designated as the antibody endpoint titer. Results were expressed as the mean ± SD (n = 5). 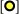 PBS; 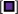 M1 VLPs; 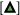 HA/M1 VLPs; 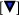 HA/M1 VLPs + sCCL28 (soluble); 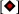 HA/M1/GPI-CCL28 VLPs.

To investigate the long-term immunogenicity of the influenza VLPs containing GPI-CCL28 *in vivo*, groups of naïve BALB/c mice were prime-boost immunized with VLPs with and without CCL28, *via* the intranasal route on day 0 and 14. Sera and mucosal washes were collected at different time-points up to month 8 after booster immunization and the resulting Aichi virus-specific IgG and IgA antibody responses were analyzed by ELISA. Animals showed higher virus-specific serum IgG endpoint titers in cVLP group when compared with the naïve groups (Fig. 1A) but titers were significantly (p < 0.05) reduced over the period of time. Serum IgG endpoint titers reached their maximum during first month and were reduced up to 10 fold by the end of the study. Interestingly, IgA levels in various mucosal lavages, especially tracheal and intestinal washes, showed significantly (p < 0.01) higher endpoint titers than other formulations. The IgA endpoint titers reached their maximum level of 12,800 in the first month, and also maintained this level over 8 months. A two-fold reduction was found at month 8 in IgA levels of the cVLP group and the mean endpoint titers reached about 6,000 both in tracheal and intestinal secretions. The same pattern occurred in the HA VLPs with sCCL28 group and showed prolonged IgA levels in various mucosal samples. The IgA levels were maintained throughout the study, suggesting the animals immunized with cVLP formulations generated potent long-lived mucosal immunogenicity (Fig. 1B–D). These data revealed that the cVLP group significantly (p < 0.01) induced long-lasting virus-specific IgA and IgG antibody levels in animals receiving only two shots of vaccine, compared to the control immunization groups.

### GPI-CCL28 enhanced HAI activity compared to other formulations

**Figure 2:**
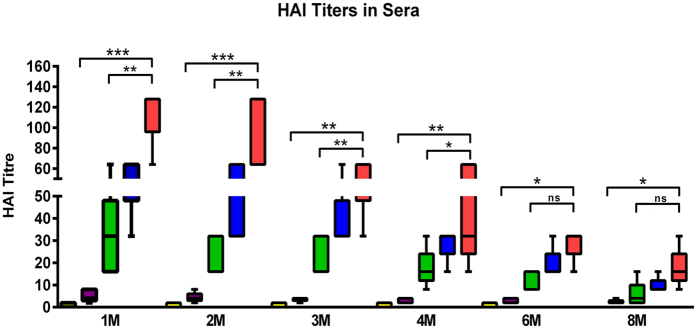
Serum HAI titers. HAI titers were estimated in the collected sera samples at months 1, 2, 3, 4, 6, 8. Sera samples were first treated with RDE by incubating overnight at 37 °C. Heat inactivated sera were serially diluted, mixed with 4 HA units of Aichi virus and incubated for 30 min at room temperature prior to adding 0.5% turkey red blood cells. Results were expressed as the minimum to maximum levels ± SD (n = 5). [Yellow] PBS; [Purple] M1 VLPs; [Green] HA/M1 VLPs; [Blue] HA/M1 VLPs + sCCL28 (soluble); [Red] HA/M1/GPI-CCL28 VLPs.

We measured the protectiveness of generated antibodies against different vaccine formulations by HAI activity. The HAI assay was used to assess functional antibodies to HA capable of inhibiting the agglutination of erythrocytes^32^. We observed that the HAI titers in sera were significantly (p < 0.05) increased in the cVLP group compared to naïve groups throughout the study. Although serum HAI titers in GPI-CCL28 group were reduced below 40 at month 8, they still maintained higher HAI levels than negative control groups. The same results were found with the influenza VLPs containing sCCL28 group. The enhancement in the HAI titers of the CCL28-containing groups suggests that the protective immunity is related to CCL28 presence in the vaccine formulations (Fig. 2).

### GPI-anchored CCL28 containing influenza VLP vaccine elicited post-challenge antibody levels and reduced viral load and inflammatory responses

**Figure 3:**
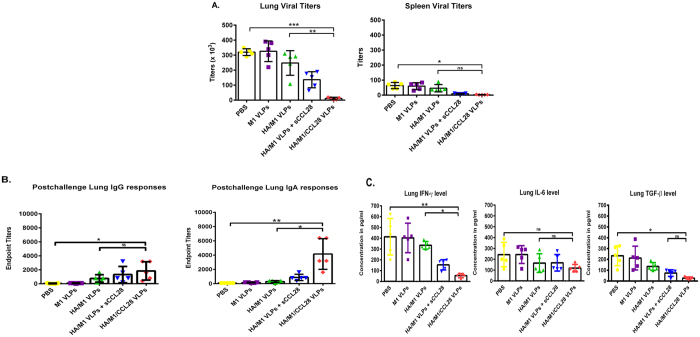
Post-challenge virus titration and immune responses in the lung. Lung and spleen were harvested on day 4 after challenge with the live Aichi virus (10 × LD_50_). Figure shows (**A**) Total virus titers (PFU/ml) in spleen and lung, (**B**) Lung IgG and IgA responses, and (**C**) Lung IFN-γ, IL-6 and TGF-β cytokines levels. Virus titers, antibody responses, and cytokine profiles were determined in the tissue extracts post-challenge. The antibody endpoint titers and cytokine levels were estimated using ELISA as mentioned in the materials and methods and viral titers were determined by standard plaque assay on MDCK cells. Results were expressed as the mean ± SD (n = 5).

To determine the continued efficacy and potency of protective immunity, we measured total viral load in lung and spleen at day 4 post-challenge. The highest lung virus titers of 3.5 × 10^5^ and 3.6 × 10^5 ^PFU/ml were found in naive and M1 VLPs group, respectively. Animals vaccinated with influenza VLPs alone and with sCCL28 demonstrated lung viral titers of 2.4 × 10^5^ and 1.4 × 10^5 ^PFU/ml, respectively. Animals immunized with VLP vaccines containing membrane-bound CCL28 were observed to have virtually non-existent levels of viral titers, 8 months after vaccination. We found significant reduction in both lung (p < 0.001) and spleen (p < 0.05) virus titers of the animals vaccinated with cVLPs. The viral titers were reduced significantly (p < 0.001) when cVLPs were compared to influenza VLPs with or without sCCL28 (Fig. 3A). Overall, mice vaccinated 8 months prior with the cVLP vaccine formulations were able to functionally eliminate the virus in both the lungs and spleen. We also evaluated IgG/IgA antibody responses and inflammatory cytokines in the lung supernatant at day 4 post-challenge. The cVLPs enhanced both lung IgG and IgA levels and showed a significant (p < 0.05) (p < 0.01) increase in their mean endpoint titers compared to the control groups. Additionally, IgA endpoint titers in the cVLP group were significantly (p < 0.01) amplified compared to IgG endpoint titers in vaccinated mice. The IgG and IgA mean endpoint titers reached 1650 and 4000 respectively, over 2 and 10 fold higher compared to the standard influenza VLP group. These results indicate the importance of CCL28 in eliciting post-challenge mucosal memory immune responses (Fig. 3B). Inflammatory cytokines, such as IFN-γ, IL-6, and TGF-β, are produced by activated macrophages and are involved in the formation of inflammatory reactions. Lower levels of these cytokines suggest increased protective immunity which inhibits the development of a full-blown infection^33^. GPI-anchored CCL28-containing vaccine formulations showed significantly (p < 0.01) reduced inflammatory cytokine levels in the lungs. IFN-γ levels in the naïve group were observed to be 400 pg/ml which was significantly (p < 0.01) reduced by 8–10 fold in the cVLP group. Similarly, the concentration of TGF-β in naïve groups was 180–200 pg/ml and levels in the cVLP group were 8-fold less in relation to negative control groups and nearly 5-fold less in relation to the influenza VLP group. We also observed a decrease in IL-6 levels in the cVLP vaccine group when compared to other formulations (Fig. 3C).

### Influenza VLP containing GPI-CCL28 vaccine contributes significantly in enhancing the recall antibody levels and HAI titers

**Figure 4:**
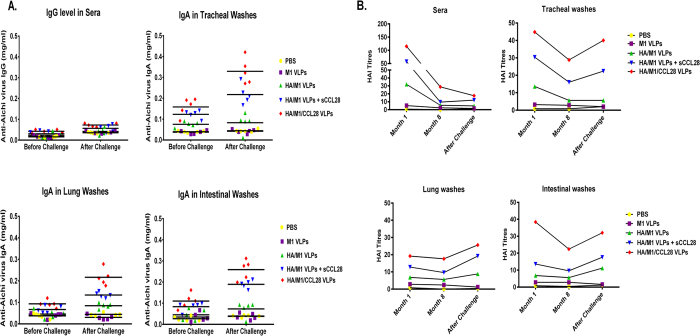
Recall antibody and HAI activity responses in sera and mucosal samples. Aichi virus-specific recall antibody responses and HAI titers were determined after challenge with the mouse adapted A/Aichi/2/1968 virus. Panel (A) represents influenza Aichi virus-specific IgG and IgA antibody levels in sera, tracheal, lung, and intestinal washes. Total IgG and IgA responses were estimated before and after challenge by quantitative ELISA using the inactivated Aichi virus as the coated antigen. HAI activity assay was performed according to the guidelines of the WHO global influenza program and the CDC protocol. Sera and mucosal lavages were collected at various time-intervals and tested for HAI activity against Aichi virus. Results were expressed as the mean (n = 5).

To determine the antibody recall responses, we evaluated Aichi virus-specific IgG and IgA before and after challenge at systemic and mucosal sites. Antibody levels and HAI titers were determined from sera and different mucosal secretions at different time-points. Virus-specific serum IgG levels were not induced after virus challenge in any of the vaccine formulations, but a slight increase in total concentrations were observed with CCL28-containing groups. Interestingly, animals vaccinated with cVLPs formulation showed a significant (p < 0.01) increase in virus-specific IgA antibody concentration generated in mucosal samples, especially in tracheal and intestinal washes, at day 4 post-challenge. After challenge with homologous virus, the amount of IgA was increased by 3–5 fold in the mice vaccinated with influenza VLPs containing membrane-bound or sCCL28. An increase in the IgA level was also observed in lung lavages, but it was lower than in tracheal and intestinal washes (Fig. 4A). Together, these data strongly suggest that CCL28 plays an important role during recall responses in inducing virus-specific IgA levels post-challenge. Similarly, one-month post-vaccination, increased HAI titers were observed in the animal groups given influenza VLPs both with and without CCL28 groups, demonstrating protective immunity. But at month 8 post-vaccination, the HAI titers in sera were reduced in all the formulations with no significant differences in HAI activity before and after challenge. Interestingly, we observed significant (p < 0.01) differences in the HAI titers of various mucosal samples before and after challenge with Aichi virus. Tracheal and intestinal washes showed higher recall HAI activity after challenge than lung washes (Fig. 4B). Thus, these data suggest that the IgA antibodies generated in these mucosal tissues stimulate long-lived and broad protective mucosal immunity against heterologous and homologous viral challenges.

### Long-term antibody production is maintained by ASCs in cVLP vaccinated animals

**Figure 5:**
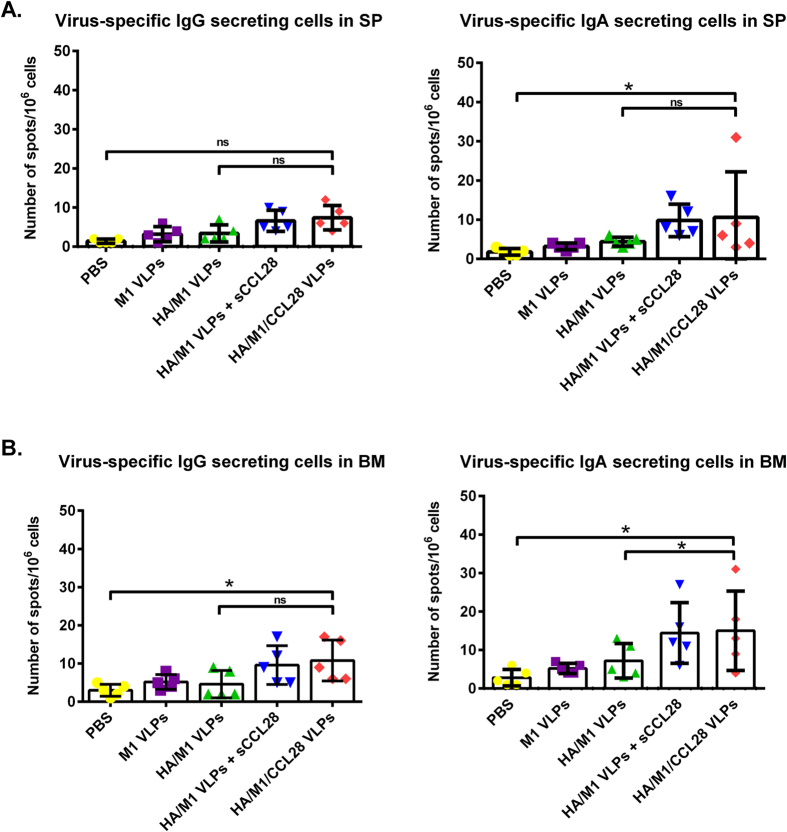
Antibody producing cell responses. Virus-specific IgG and IgA ASCs were determined from spleen, and bone marrow at day 4 post-challenge using ELISPOT. To determine the number of antibody producing cell responses *in vitro*, bone marrow and spleen cells were cultured in multiscreen 96-well filtration plates, coated with inactivated Aichi virus, and levels of total IgG and IgA ASCs were determined using HRP-conjugated anti-mouse IgG/IgA antibody. Results were expressed as the mean ± SD (n = 5).

Antibody recall responses play a critical role in providing long-lived protective immunity^34^. Thus, we assessed ASC levels at day 4 post-challenge with Aichi virus in spleen and bone marrow using ELISPOT assay. Although animals immunized with cVLPs showed weak IgG secreting cell responses, post-challenge IgA ASC levels were significantly (p < 0.01) increased in bone marrow and spleen (Fig. 5A). The increase in the number of spots was highest in the groups containing CCL28, while control groups showed low ASCs (Fig. 5B). These data demonstrate that CCL28-containing vaccine formulations induce long-lived cells capable of producing virus-specific antibodies.

### CCL28-containing influenza VLPs confer long-term protection in vaccinated animals against homologous and drifted H3N2 influenza viruses

**Figure 6:**
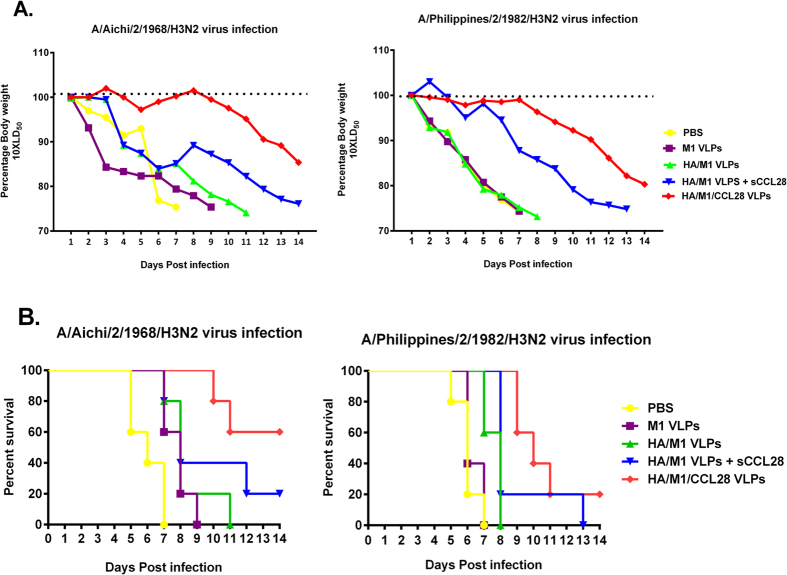
Protective efficacy of vaccine formulations. 8-months post-vaccination, animals were challenged with 10 × LD_50_ of mouse adapted A/Aichi/2/1968 or A/Philippines/2/1982 H3N2 viruses. Vaccinated and control groups were monitored up to 14 days for body weight changes, fever, hunched posture, illness features, and mortality. Figure showed (**A**) Body weight changes, and (**B**) Survival rates in Aichi, and Philippines challenged mice. Weight loss exceeding 25% was used as the experimental endpoint, at which mice were euthanized according to IACUC guidelines. Body weight changes are displayed as the mean; representative of one experiment. The survival differences were evaluated by the Log rank Mantel-Cox test (n = 5).

We challenged vaccinated animals with lethal doses (10 × LD_50_) of A/Aichi/2/1968 or A/Philippines/2/1982 H3N2 influenza viruses to determine the level of protection conferred by vaccine formulations, 8-month post-vaccination. Body weight changes were also measured on a daily basis after a lethal viral challenge, with an endpoint of 25% body weight loss, below which the mice were euthanized. Influenza VLPs containing sCCL28 or GPI-CCL28 showed 20% and 60% long-term protection with about 22% and 16% body weight loss, respectively against homologous influenza (Aichi) virus (p < 0.05) infection. All animals in the negative-control and standard influenza VLP groups rapidly lost their body weight and were euthanized when their body weight loss exceeded 25%. During Philippines viral challenge, animals vaccinated with cVLPs showed a 20% cross-protection after 8-month post-vaccination, while none of the mice in the naive, M1, HA/M1, and even in the sCCL28 containing VLP groups survived (Fig. 6A,B). The mice in the cVLP vaccination group showed lesser body weight loss for the first week post-challenge when compared with negative groups. The surviving mice maintained body weight levels greater than 75% of their initial weight throughout the challenge study. We observed the surviving animals for two more weeks after the completion of challenge studies and found that the surviving mice regained their body weight and showed no clinical signs of infection.

## Discussion

Influenza is a highly contagious and acute respiratory illness caused by the influenza virus. The influenza virus frequently undergoes antigenic variation resulting in Worldwide epidemics or local outbreaks^35^. Available vaccines induce strain-specific immunity; highly protective against homologous but less effective against drifted/shifted virus strains^36^. Other challenges affecting current vaccination methods are the cost-effectiveness and short duration of protective immunity^37^.

Because influenza targets the respiratory tract, mucosal antiviral responses such as local antibodies generated by recruited B lymphocytes, or alternatively transudated into the mucosa from systemic circulation, are thought to contribute to providing long-term mucosal cross-protective immunity. The mucosal antibodies are present at the entry site of the virus, possibly neutralizing the virus and thus preventing infection^14^. In the current study, we demonstrated that influenza VLP vaccines containing GPI-anchored CCL28 induced long-lasting protective immunity against infection with either homologous or heterologous H3N2 viruses. Our previous study also showcased that GPI-anchored CCL28 in influenza VLPs act as a strong adjuvant/immunostimulator at systemic and mucosal sites when immunized in mice *via* the intranasal route^31^. Thus, the capability for inducing long-lived protective immunity would be an important added strength to this approach.

In humans, secretion of IgA by the mucosal immune system accounts for ~70% of the body’s total antibody production. The importance of mucosal immunity in mounting a defense against influenza virus has been suggested by many research findings^38^,^39^. Mucosal antibodies have also been previously implicated in increasing resistance to severe influenza infection^40^. Data acquired from volunteers infected with wild type or attenuated influenza virus indicate that IgA antibodies in particular were elicited in the respiratory secretions and were able to confer resistance to infection and illness^41^. Therefore, strategies to protect mucosal surfaces, including vaccines designed to generate protective immunity, are needed. To better understand how to construct a protective mucosal vaccine that could protect the earlier mucosal targets of influenza infection, we focused on CCL28, a chemokine that plays an important role in the migration of IgA ASCs in the mucosal sites^27^. Chemokines allow for the fine tuning of the organization of the mucosal immune system and also enable the cells of mucosal immune system to relocate as needed in response to antigenic changes, infection by pathogens, and other type of insults. Results presented herein demonstrate that immunization of mice with Aichi HA expressing VLPs in the presence of GPI-anchored CCL28 results in the induction of prolonged virus-specific antibody endpoint titers, especially IgA responses in various mucosal secretions. It should be noted that the decline in the IgA and IgG antibody responses in systemic and mucosal compartments was significantly lower in the cVLP group than in the control groups. These results suggest that systemic and mucosal antibody responses are efficiently modulated by the GPI-CCL28 on the cVLPs. CCL28 chemokine works as a innate immune molecule and are also helpful in the migration and localization of T and B cells. Simultaneously, CCL28 may be involved in antigen processing and presentation, and play an important role in the maturation of dendritic cells. However, this concept remains to be determined. Thus, the CCL28 chemokine may work as a connecting link between innate and adaptive immunity, and assist in the generation of strong antigen (co-delivered with the CCL28) -specific immune responses. As our vaccine composition contains influenza HA and GPI-anchored CCL28 co-incorporated into the same cVLP, which leads to the delivery of antigen and an immune stimulator to the same immune cells and may contribute a robust increase in the influenza specific immune responses in the cVLP group. We found that the IgA immune responses in various mucosal secretions were sustained up to 8 months while serum IgG levels were reduced over the same period. This might be explained by the characteristic property of CCL28 to particularly attract IgA but not IgG and IgM ASCs expressing CCR3 and CCR10 chemokine receptors^28^,^29^,^30^. The CCR3/CCR10 and CCL28 interaction orchestrates both the migration and recruitment of IgA secreting plasma blasts and plasma cells into various mucosal tissues, which may leads to a prompt mucosal, but not systemic antibody response as such^26^,^27^.

Protective immunity depends not only on preformed antibody and effector T cells, but more importantly on the establishment of a population of lymphocytes that mediate long-lived immunological memory. In the current study, we observed that the use of CCL28 significantly improved the vaccine efficacy and antigen-specific long-lasting immunogenicity. The cVLP vaccine formulation showed increased memory antibody responses at mucosal sites with high cross-protection when compared to the negative groups. GPI-anchored CCL28 in influenza VLPs may enhance both the signal strength and the length of exposure of antigens in circulation. Simultaneously, CCL28 may be contributing to both clonal expansion and clonal differentiation in B cells and/or may be increasing antibody affinity due to somatic hypermutation and selection of antigens in germinal centers^42^. However, the underlying mechanism remains to be determined and requires continued investigation.

Antibody and T cell responses are key metrics by which we can characterize the magnitude and functionality of immune memory while other aspects such as antibody avidity, neutralization breadth and potency, and polyfunctionality have also been associated with protection. To check the efficacy of our vaccine formulations, we also evaluated other immune parameters such as HAI activity, recall responses, viral titers, lung antibody and inflammatory cytokine levels, and body weight changes with animal survival after challenge. We characterized the HAI activity in sera and mucosal samples for all the formulations against Aichi virus at different time-points. Serum HAI titers were reduced periodically but HAI titers in various mucosal washes remained persistent throughout the study, especially in cVLP group. As HAI activity is the most established associate with the vaccine protectiveness, these results also demonstrated a direct link with constant levels of generated antibodies^42^. Despite extensive clinical and pathological descriptions of influenza, our understanding of the mechanisms of disease development with respect to the generation of various cytokines during infection is still incomplete^43^. Various findings have shown that ‘how’ cytokines exacerbate inflammation and play a central role in the resolution of the infection by clearing the viral load of influenza deserve attention^38^,^43^. We measured various inflammatory cytokines and virus titers in the supernatant at day 4 post-challenge. The lower levels of inflammatory cytokine (IFN-γ, IL-6, and TGF-β) and viral titers in the lung supernatant of the cVLPs groups also implies a significant role of inflammatory cytokines in the clearance of virus.

For efficient vaccines, it is required to induce a sufficiently strong and rapid immunological recall response by memory cells to provide complete protection^38^. In order to examine the efficacy of recall response induced by vaccination, animals were challenged at 8-month post-vaccination, and their antibody responses and HAI titers were determined at day 4 post-challenge. Interestingly, we found a robust increase in IgA levels and HAI activity in mucosal samples of the animals immunized by GPI-CCL28 containing VLPs. In contrast, the animals vaccinated with influenza alone or with sCCL28 showed significantly lower recall responses when compared to cVLP vaccines^44^. During the Aichi virus survival study, we found higher survival rates and less body weight changes in the cVLPs group. However, the animals in the cVLP group showed lower protection against the heterologous virus challenge. The animals immunized with influenza VLPs alone or with sCCL28 showed low homologous protection, while no animals survived the Philippines (drifted) virus infection. Whether the lower memory response is connected to survival or indicates a mechanistic difference in the induction of systemic and mucosal memory responses to antigen remains to be determined.

Taken together, elevated and prolonged IgA antibody responses in mucosal locations, high HAI titers, low viral load and inflammatory cytokine secretion, strong mucosal recall responses, and high survival rates were found with cVLP vaccine formulations. These results strongly suggest that GPI-anchored CCL28 in cVLP formulations elicited robust to modest protective immunity against Aichi and Philippines H3N2 viruses, respectively through sustained antibodies secretion and rapid recall responses at mucosal compartments.

## Conclusion

The current study supports the importance of GPI-anchored CCL28 in the influenza VLPs vaccine formulation in inducing effective, long-lasting mucosal immune responses with strong to moderate protective immunity against homologous and heterologous H3N2 influenza viruses, respectively through the induction of IgA levels in the various mucosal compartments. The results demonstrate that GPI-anchored CCL28 containing influenza VLPs are effective vaccines when administered *via* an intranasal route, which may provide a useful methodology for developing a platform for the next-generation influenza vaccines.

## Materials and Methods

### Ethics statement

This study was carried out in strict accordance with the recommendations found in the Guide of the Care and Use of Laboratory Animals of the National Institutes of Health (NIH). All animal studies were approved by the Institutional Animal Care and Use Committee (IACUC). 6–8 week healthy BALB/c female mice were purchased from Jackson Laboratory, and housed in the animal facility. Immunization and bleeding were performed under mild anesthesia that was induced and maintained with ketamine hydrochloride and xylazine, and all efforts were made to minimize pain.

### Viruses

Influenza virus, A/Aichi/2/1968 (H3N2) was harvested from the allantoic fluids of infected eggs and purified by using a discontinuous sucrose gradient (15%, 30%, and 60% layers) and ultracentrifugation at 96000× g for 60 min. The purified virus was inactivated using formalin at a final concentration of 1:4000 (v/v). The inactivated Aichi virus was used as an antigen for enzyme-linked-immunosorbent assay (ELISA). For challenge experiments, mouse-adapted A/Aichi/2/1968 and A/Philippines/2/1982 (H3N2) viruses were prepared as lung homogenates of infected mice^45^. The LD_50_ (Lethal dose inducing 50% mortality) and TCID_50_ (Tissue culture infectious dose infecting 50% cells) of these strains were determined by infection of mice and cells, respectively, with serial viral dilutions and calculated by the Reed and Muench method^46^.

### Cell-lines

*Spodoptera frugiperda* insect cells (SF9) (ATCC, Manassas, VA, USA) were maintained in serum-free SF900II medium (Life Technologies, Carlsbad, CA, USA) while *Madin Darby Canine Kidney* cells (MDCK) (ATCC, Manassas, VA, USA) were grown and maintained in Dulbecco’s modified Eagle’s medium (DMEM) (Corning Inc., Corning, NY, USA).

### Preparation and characterization of influenza and cVLPs

The GPI-anchored CCL28 gene was constructed with the fusion of honeybee melittin and murine CD59 GPI-anchor as described previously^31^. M1, HA/M1, and cVLPs containing HA and M1 proteins derived from influenza Aichi virus co-incorporated with GPI-anchored CCL28 were produced using a recombinant baculoviruses (rBV) expression system^47^. The protein concentration in all VLPs was quantified with a protein assay kit (Bio-Rad, Hercules, CA, USA), and the expression of M1, HA, and CCL28 in VLPs were determined by Western blot using specific antibodies. Total CCL28 and HA content in the produced VLPs were estimated by a quantitative ELISA using recombinant CCL28 (Prospoec, Brunswick, NJ, USA), and Aichi HA (Sino Biological Inc., North Wales, PA, USA) as the calibration standards, respectively. The biological activities of HA protein and CCL28 in VLPs were determined by hemagglutination assay and *in vitro* chemotactic activity towards the cells expressing CCR3 and CCR10 chemokine receptors, as described previously^31^. These various physical and biological assays showed the integration and functional stability of HA and CCL28 in the VLPs. The results confirmed high incorporation of respective proteins with their functional stabilized structure in the VLPs^31^. Our vaccine strategy was to incorporate Aichi HA and GPI-CCL28 as antigen and adjuvant respectively into the same cVLP structure capable of delivering both components to the same cells, potentially enhancing antigen-specific immunogenicity.

### Immunization and sample collection

Female BALB/c mice aged 6–8 weeks were used for VLP immunization. Groups of mice (n = 5) were intranasally primed at day 0 with a boost of the same formulations at day 14. Animals were immunized with 1 μg of Aichi HA and 0.5 μg of CCL28 (membrane-bound or soluble) per mouse during each immunization. Animals were immunized with the same amount of CCL28 in both the membrane-bound and soluble forms^31^. We compared the immune responses of cVLPs containing HA and GPI-CCL28 to that from influenza VLPs without CCL28, or physically mixed with sCCL28. Phosphate buffered saline (PBS) and M1 VLPs were used as negative control groups. For measuring long-lasting immune responses, sera and mucosal wash (tracheal, lung, and intestinal) samples were collected at month 1, 2, 3, 4, 6, and 8 post-vaccination. Sera were collected from the clotted blood by centrifugation at 1500× g for 10 min at 4 °C. After the collection of blood samples, animals were sacrificed and tracheal, lung, and intestinal lavage samples were collected by repeated flushing of the respective cavities with 1 ml of ice cold lavage medium (0.9%, *w/v*, NaCl; 0.05%, *v/v*, Tween 20; 0.1%, *w/v*, NaN_3_; and 1 mol/dm^3^ PMSF). Sera and mucosal washes were stored at −80 °C till further use.

### Evaluation of antibody responses and HAI titers

Antigen-specific antibodies in sera and mucosal washes were evaluated on 96-well flat bottom microtiter plates (Nunc-Immuno Plate Maxisorp; Nunc Life Technologies, Basel, Switzerland) coated with inactivated Aichi virus as described previously^31^. The optical density at 450 nm (OD_450_) was read with an ELISA reader (BioTek, Winooski, VT, USA). The highest dilution which gave an OD_450_, two fold higher than that of the naive group without dilution, was designated as the antibody endpoint titer. For HAI activity, receptor-destroying enzyme (RDE) (Denka Seiken, Campbell, CA, USA) treated sera and mucosal samples were incubated with 4 HA units of Aichi virus prior to the addition of 0.5% turkey red blood cells^12^. The HAI titer was determined as the dilution of sample that inhibited influenza virus agglutination of erythrocytes. Aichi virus-specific antibody levels and HAI titers were also evaluated before and after challenge to check the recall responses at day 4 post-challenges.

### Viral titers, antibody and inflammatory cytokine responses

Lung and spleen viral titers at day 4 post-challenge were determined by counting plaques formed on the MDCK cells. The lungs and spleen were removed and homogenized in 1 ml of PBS containing antibiotics. The tissue homogenates were centrifuged, and the supernatants were tested for total viral load by standard plaque assay on MDCK cells and results were shown as PFU/ml. Simultaneously, inflammatory cytokines *i.e.* interferon gamma (IFN-γ), interleukin-6 (IL-6), and tumor growth factor beta (TGF-β) (BD Pharmingen, San Diego, CA, USA), and IgG/IgA levels (Southern Biotech, Birmingham, AL, USA) in lung supernatant were also analyzed by ELISA using specific paired antibodies^48^.

### Post-challenge virus-specific antibody secreting cell response estimation

Virus-specific IgG/IgA ASCs were determined from bone marrow and spleen at day 4 post-challenge using ELISPOT. Inactivated Aichi virus was used to coat multiscreen 96-well filtration plates (Millipore, Bedford, MA, USA). Freshly isolated cells from bone marrow and spleen (1 × 10^6^) cells were added to each well and incubated for 12–15 h at 37 °C with 5% CO_2_. Using HRP-conjugated anti-mouse IgG/IgA antibody (Southern Biotech, Birmingham, AL, USA) and 3–3′-diaminobenzidine tetrahydrochloride (DAB) (Research Genetics Inc., Huntsville, AL, USA) substrate, color was developed, and counting of spots was performed using an ImmunoSpot ELISPOT reader (BioSys, Miami, FL, USA)^49^.

### Virus challenge studies

For virus challenges, vaccinated animals were lightly anesthetized and challenged with 10 × LD_50_ of A/Aichi/2/1968 or A/Philippines/2/1982 H3N2 viruses in 20 μl of PBS, administered into both nostrils. Mice were monitored daily for morbidity and mortality for 14 days after challenge. Mice that lost more than 25% of their original body weight were recorded as dead and necropsied.

### Statistical analyses

The data for antigen-specific IgG/IgA levels, ASCs, and HAI titers were analyzed by paired student t-tests and compared by parametric one-way ANOVA analysis of variance by ranks. Virus-titers, antibody levels, and inflammatory cytokines in lung supernatant were also analyzed by paired student t-tests or one-way ANOVA tests. n = 5 mice per group and the results were expressed as mean ± standard deviation (SD). Survival differences were evaluated by Log rank Mantel-Cox test. Levels of significance (p value) were compared between the PBS, and influenza VLP groups with the cVLP group. Tests were performed using GraphPad Prism 7 software (San Diego, CA, USA). p values of <0.05 (p < 0.05) were considered to be statistically significant. *p < 0.05; **p < 0.01, ***p < 0.001, ****p < 0.0001.

## Additional Information

**How to cite this article**: Mohan, T. *et al*. Chimeric virus-like particles containing influenza HA antigen and GPI-CCL28 induce long-lasting mucosal immunity against H3N2 viruses. *Sci. Rep.* **7**, 40226; doi: 10.1038/srep40226 (2017).

**Publisher's note:** Springer Nature remains neutral with regard to jurisdictional claims in published maps and institutional affiliations.
